# Assessment of Milk Contamination, Associated Risk Factors, and Drug Sensitivity Patterns among Isolated Bacteria from Raw Milk of Borena Zone, Ethiopia

**DOI:** 10.1155/2022/3577715

**Published:** 2022-06-20

**Authors:** Alqeer Aliyo, Zelalem Teklemariam

**Affiliations:** ^1^Department of Medical Laboratory Science, Institute of Health, Bule Hora University, Bule Hora, Ethiopia; ^2^Department of Medical Laboratory Sciences, College of Health and Medical Science, Haramaya University, Harar, Ethiopia

## Abstract

**Background:**

The contamination of raw milk depends on the number and type of organisms that can cause health risks, which can be judged by the presence of microorganisms and bacterial pathogens. This study evaluated bacterial contamination, the risk factor, and drug sensitivity patterns.

**Methods:**

A cross-sectional study was carried out on conveniently selected 95 milk producers. Data were collected using the structured pretest questionnaire and the observation control list. Subsequently, 15 to 20 ml of milk samples were taken for laboratory analysis. The milk samples have been diluted and continuously inoculated on the number of standard plates and the blue Eosin methylene germs for the total number of bacteria and coliforms counted. Biochemical and drug sensitivity tests have been done. The version 21 Statistical Package for the Social Sciences was used for analysis. Analysis of the associated factors using binary logistical regression analysis and a *P* value less than 0.05 was considered to be statistically significant.

**Results:**

The total bacteria (TBC) and coliform count (CC) average with the standard deviation were 7.57 ± 0.83 log10 and 6.54 ± 1.53 log10 CFU/mL, respectively. The prevalence of raw milk contaminated with TBC and TCC was 84 (88.4%) and 75 (78.9%), respectively. Lack of handwash practice before milking (AOR = 2.4 [95% CI: 0.35–16.4]) and using unclean milk containers (AOR = 7.47 [95% CI: 0.0023–28.64]) were found to be significantly associated with bacterial contamination of raw milk. The bacteria isolated were *E*. *coli* (30.7%), *Staphylococcus aureus* (16.7%), and *Salmonella* spp. (1.2%). Among isolated bacteria, 76.3% were extensive drug resistant, 13.2% were multidrug resistant, and 2.6% were resistant to all drugs tested in the current study.

**Conclusion:**

Guaranteed appropriate hygiene exercise during time of milking and clean containers reduced milk contamination. Doctors should consider resistance to drugs during the treatment of patients with milk disease.

## 1. Introduction

Raw milk is a dairy product that promotes body tissue growth and maintenance by providing the necessary nutrients like proteins, energy, minerals, and vitamins [1]. Biologically important macromolecules such as proteins and casein in milk have been proven to be crucial for biochemical physiological functions which have an important effect on human health and metabolism. The immunoglobulin class in the milk is significant to protect newly born against different types of diseases [2].

Milk and other dairy products are useful for nourishing the pastoral community in Ethiopia. In lowland parts of the country where livestock rearing is the main occupation, the majority of social groups consume raw milk [3]. Raw milk is sold directly to consumers without low-temperature sterilization by producers and informal markets. Such a practice is a means of transmitting pathogens mediated by milk to humans, causing milk deterioration in the time of production, manipulation, transport, and transformation [4].

Milk contaminated with pathogenic bacteria is a major cause of foodborne disease, which is a serious health problem for millions of people in the world. Food-related diseases causing mortality or other complications due to contaminated milk increase every day and create a significant burden on the healthcare system [5]. Among food poisoning bacteria, salmonella causes the most widespread diseases in the world and is estimated at 1.3 billion gastroenteritis and 3 million deaths worldwide [6]. Similarly, food poisoning caused by *Staphylococcus aureus* (SFP) is also the most widespread cause of gastroenteritis in the world [7]. In addition, *E*. *coli* O157 : H7 is the other most important food mediator, causing human diarrhea, hemorrhagic colitis, and hemorrhagic therapy syndrome. Clear links between consuming raw milk and human illness were found in the cases of *Campylobacter* spp.*, Salmonella* spp., *Brucella melitensis*, and *Mycobacterium bovis* [8].

Food-associated diseases are responsible for 33 to 90% of the deaths of children in Africa, and represent serious problems of the continent [9]. In developing countries, particularly Ethiopia, milk is an important source of foodborne diseases and other infectious diseases. This happens when the production of milk and various dairy products occurs under unsanitary conditions and bad production practices [10].

Overdose, misuse, and prolonged use of drugs for treatment of animals and humans lead to an alarming increase and distribution of antimicrobial resistant bacteria. This is worsening the clinical scenario and is one of the greatest medical challenges of our time. It is also the reason for low cure rates, loss of human life, and animal life, and animal milk products [11].

Generally, raw milk and dairy product contamination control is not done on a daily basis in Ethiopia [12]. This situation increases the contamination of raw milk among pastoral communities of the country [13]. Likewise, the pastoral community of Borena zone, Gomole district, provides milk directly to consumers without taking appropriate measures. Consequently, this study was carried out to assess milk contamination, the related factors, and drug sensitivity testing in Borena zone, Gomole district, pastoral community, Southern Ethiopia.

## 2. Methods

### 2.1. Study Setting and Period

This study was conducted in the Oromia regional state, Borena zone, Gomole district, Southern Ethiopia from March 1 to April 30, 2019. The Gomole District is one of the 10 districts in the Borena zone and 525 km South of Addis Ababa, located on the Addis Ababa-Moyale highway. The Gomole district has 67,798 people in total and more than 90% of the population is in pastoral communities. This district is dry and semiaged, and the average annual daily temperature varies from 17°C to 30°C with a bimodal season. According to Animal Statistics in 2018, the region has 37,000 cows, 14,890 camels, and 108,222 goats. Raw milk and dairy products are the main export products to neighboring areas and cross the border to Kenya.

### 2.2. Study Design and Population Selection

A cross-sectional study design was used on the conveniently selected study participants in the Gomole district of Borena zone. Raw milk not from selected kebele of Gomole district and their owners were excluded from the study. During the study period, milk from producers in selected Kebeles was included in the study, while the milk from vendors and milk imported from outside the selected kebeles was excluded from the study.

### 2.3. Sample Size and Sampling Method

Using the double population proportion formula, the sample size was calculated by Epi Info Version 7.2.1.0 [14] with the following assumptions: power of the study = 80%, with a 95% confidence level, the ratio of unexposed : exposed is 1 : 1 including 15% nonrespondent rate. The final sample size obtained was 97. Out of 14 kebeles (locations) in the Gomole district, four kebeles were initially selected using a lottery method. The milk producers/owners in each kebele were proportionally allocated, and then participants were selected using a convenient sampling technique.

### 2.4. Data Collection Methods

The pretested structured questionnaires and observation checklists were used to collect data through face-to-face interviews with the participants. The questionnaire contains sociodemographic characteristics, such as age and sex, and other factors that can contaminate raw milk during observation to gather data on conditions around milk, containers, and the surrounding environment (S1 file). Then, aseptically collected 15–20 milliliter (mL) milk samples were directly collected from participant containers and placed into sterile screw cups. The collected samples were labeled, kept in a 4°C ice box, and transported to the microbiology laboratory for analysis.

### 2.5. Bacteria Enumeration and Isolation

Total bacteria number and coliforms were counted by culturing on standard plate count agar and eosin methylene blue agar (EMBA), respectively. 1 mL of milk was transferred to a sterile test tube which contained 9 mL of sterile peptone water. The mixture was then serially diluted up to 10^−7^ . One milliliter (mL) of diluted milk portion was taken and inoculated into sterile standard plate count agar media and eosin methylene blue agar (EMB) media (Oxoid, Basingstoke, UK) (S2 file). The inoculated plates were grown at 35°C overnight [15]. The plate growth colonies between 25–250 colony-forming units per milliliter (CFU/mL) of sample were taken to determine the total bacteria quality [16]. Total bacteria count became decided as the total number of CFU per milliliter of milk sample that was calculated by using this formula. CFU/mL = mean of enumerated number of colonies/Dilution factor × Volume plated. The counted TBC was determined according to the East African standard milk samples. The raw milk was noncontaminated if the bacteria count was less than 2 × 10^6^ CFU/mL (<6.3log10) and contaminated if >2 × 10^6^ (>6.3log10) CFU/mL [15]. The total coliforms were determined by counting green metallic sheen dark centered nucleated colonies which appeared on the plates after incubation [16]. The result was judged by the East African standard with raw milk that had total coliforms count <50,000 (<4.7 log_10_) CFU/mL was accepted as noncontaminated [17].

*Escherichia coli* bacteria were isolated from coliform contaminated raw milk by culturing on eosin methylene blue agar (EMBA) plates. Then one or two typical greenish metallic sheen suspected colonies were examined with the Gram staining technique, and Gram-negative bacilli isolated were determined biochemically [18]. For *Salmonella* species isolation, one millileter of raw milk portion was added into 9 mL of lactose broth and incubated at 35°C overnight. Then, one milliliter of diluted milk was inoculated in to 10 mL selenite F broth for preerichment and incubated at 35°C overnight. From enrichment media, a loop full of sample was inoculated on Xylose-lysine decarboxylase (XLD) and incubated at 35°C overnight (S2 file). After incubation, nonlactose fermenting *Salmonella* species suspected colonies were taken from XLD media and inoculated into nutrient agar plate and incubated at 35°C overnight. Biochemical tests were done on a pure single colony isolated from plate agar [13]. Regarding *Staphylococcus aureus* isolation, 0.1 mL aliquots from stored dilution of total bacteria contaminated samples were inoculated into mannitol salt agar (MSA) plates and incubated at 35°C overnight. When one or two yellowish suspected colonies grew on the media, pure colonies were transferred from MSA plate into nutrient broth (NB) tubes and incubated at 35°C overnight. Then one loop full of suspension was inoculated onto nutrient agar and incubated at 35°C overnight. Then Gram staining examination, catalase, and coagulase test were performed on pure isolated colonies. The colonies formed clotting/clumping with coagulase test were considered as *Staphylococcus aureus* (S2 file) [19].

### 2.6. Drugs Sensitivity Test

The bacteria isolates underwent drug sensitivity test which was carried out by the diffusion technology of the Kirby–Bauer disc modified by clinical and laboratory standards (CLSI). Three to five pure colonies of bacteria were collected and added into the tube that had 4 to 5 mL physiological saline and were gently mixed in the solution to adjust homogeneous turbidity with McFarland 0.5 standard. The sterilized swab cotton in the prepared suspension was dipped and sprayed on the whole surface of Mueller–Hinton Agar (UK) in accordance with the directives of the Standard Clinical and Laboratory Research Institute [20]. Then, fixed concentrations of drug discs were used for studies. The antimicrobials such as chloramphenicol, ampicillin, tetracycline, ciprofloxacin, and gentamycin were selected based on the possibility of using prescriptions for the treatment of animal infected with bacteria and the frequency of use in the country [21]. The discs were placed into the Muller–Hinton agar and incubated at 35°C overnight. The transparent inhibition zone was generated and dissemination of the bacteria after incubation, was determined and interpreted [20].

### 2.7. Quality Control

The quality of the data obtained is guaranteed by following the standard procedure at each stage of the task. The questionnaire on this study was adopted in previous studies and was developed in the region. The questionnaire was pretested in advance with the completeness, and the adequacy of the regional context was tested on 5% of the participants at the District of Fichwa. Some questions have been set according to the results before the test. Daily data collectors, supervisors, and main researchers confirmed the questionnaire on site after data collection. The calibrated equipment was used to measure the reagents and other materials before using them in the process. The quality of the reagents, antibiotic discs, and disinfection solutions was guaranteed according to the manufacturer's management, and the expiration date has been confirmed. Before the distribution, the antibacterial disc was held at room temperature for 1 hour. The culture medium was applied and sterilized according to the manufacturer's directives, and then the sterile media were inspected by incubating 3 to 5% of the lot at 35°C overnight. Collection of American Culture of type (ATCC) reference species such as *E*. *coli*, *S*. *aureus*, and *S*. *typhimurium* were used for culture quality check.

### 2.8. Data Analysis

The data were checked for completeness and consistency, and double data entry was made using Epi-data version 3.5.1 software. Then, the data were exported to SPSS version 21 for further analysis. The total bacterial and coliform count results were interpreted as direct and converted into Log10 CFU/mL. Descriptive statistics such as percentage and frequency were computed, and the mean with standard deviation or median with interquartile range was used to summarize the continuous variables accordingly. Different types of graphs or charts and tables were used to present the data. Bivariate and multivariate logistic regression analyses were used to identify factors associated with raw milk contamination. The variables in bivariate analysis with *p* values less than 0.25 (*p* < 0.25) were included in multivariate analysis. Multivariate logistic regression was performed to control for potential confounders. The model fitness was checked using the Hosmer–Lemeshow goodness-of-fit test. Finally, the strength of associations between outcome and determinant variables was expressed using adjusted odds ratios (AORs) with 95% confidence intervals, and the significance of associations was declared at a *p* value of less than 0.05.

## 3. Results

### 3.1. Questionnaire and Observational Survey

In total, 95 participants signed up for this study, and the response rate was 97.9%. The respondents' ages were between 19 and 67 years old, with an average and standard deviation of 38.1 ± 11.4. In terms of sex, most of the research participants 77 (81%) were female. Regarding education status, 84 (88.4%) could not write and read, only 4 (4.2%) went to primary school. On the other hand, 82 (86.3%) respondents were never trained on milk handling, while 74 (77.9%) among the participants in the research did not know the diseases transmitted via milk contamination (Table 1).

Among interviewed participants, the majority of milk producers 72 (75.8%) were using plastic containers, more near half of respondents 52 (54.7%) transported with motorcycles, and 46 (48.4%) transported milk for less than two hours (Table 2).

Regarding hygienic practice observations, approximately 77 (81.1%) participants used containers with poor cleanliness, 45.3% had good personal hygiene, and 41.2% kept the milk under cool conditions (Table 3).

### 3.2. Total Bacteria and Coliforms Enumeration

The mean total bacterial count (TBC) was 7.57 ± 0.83 log_10_ (3.7 × 10^7^) CFU/mL, and the mean total coliform count (TCC) was 6.54 ± 1.53 log_10_ (3.2 × 10^6^) CFU/mL (Table 4).

### 3.3. The Prevalence of Raw Milk Contamination and Bacterial Isolates

The proportions of TBC- and TCC-contaminated milk were 84 (88.4%) and 75 (78.9%), respectively. Bacteria isolated from contaminated milk were *Salmonella* spp. (1.2%), *S*. *aureus* (16.7%), and *Escherichia coli* (30.7%) (Table 5).

### 3.4. Risk Factors Associated with Milk Contamination

Logistical regression analysis has shown that water supply sources for equipment cleaning, knowledge of milk collapse, handwashing practices before milking, and containers of milk were less than 0.25. However, multivariate logistical regression analysis, lack of hands wash before milking (AOR = 2.4 (95% CI: 0.35–16.4)), and unclean milk containers (AOR = 7.47 (95% CI: 0.002–28.64)) was significantly linked to bacterial contamination of raw milk (Table 6).

### 3.5. Antimicrobial Test of Bacteria Isolates

Among isolated bacteria both *E*. *coli* and *S*. *aureus* were sensitive to ciprofloxacin. The isolated *E*. *coli* was highly resistant to ampicillin 19 (79.9%). From bacteria isolates, *Salmonella* spp. showed high resistance against the drugs tested in the current study (Table 7).

The prevalence of multidrug resistance (MDR) and pandrug resistance of isolated bacteria were 13.2% and 2.6%, respectively (Table 8).

## 4. Discussion

In this study, both total bacteria and coliform enumeration were performed by culturing on appropriate media. The total bacterial count mean (TBC) was 7.57 ± 0.83 log_10_ (3.7 × 10^7^) CFU/mL. This result was comparable with a reported study done in Yabelo (8.149 log_10_) [22]. In contrast, this result was greater than reported from Iran (1.03 × 10^6^ CFU/mL) [23] and Dire Dawa (6.76 Log_10_ CFU/mL) [24]. On the other hand, the mean of total coliform count (TCC) was 6.51 ± 1.53 log_10_ (3.2 × 10^6^) CFU/mL. This finding was similar to the studies done in Yabelo (6.323 ± 0.028 log_10_) [22], Ethiopian Eastern (7.32 ± 0.07 log_10_) [25], and Hawassa (6.52 log_10_ CFU/mL) [15]. In contrast, this result was greater than reported from Kenya, Isiolo (4.00 ± 0.66 log10 CFU/mL) [26], Tanzania (2.8 × 10^4^ CFU/mL) [27], and Ethiopia, Mersa (5.15 log_10_ CFU/mL) [28]. The difference in research is the seasonal variations, surrounding temperature, hygienic status of milkers, and the contamination of bacteria from milk containers, particularly during transportation [10].

Milk contaminated by TBC was 88.4%. This result was comparable with the study done in Tanzania (90%) [27]. However, it was greater than the study done in Adigrat (60%) [29], but less than Boraena Abaya (99%) [13]. The higher the TBC, the less the quality of milk, shelf life, and the milk nutritional content can be reduced, and toxic metabolites produced by other organizations that are growing can threaten the health of consumers [19].

The prevalence of isolated *Ensercheia coli* was 30.7%. This finding was greater than the study conducted in Tanzania (3.5%) [27], Zambia (13%) [30], Abaya Borena (12.9%) [13], Hawassa (8.8%) [31], and Mekelle (17.6%) [32]. However, the present study finding was lower than that of Tanzania, Arusha (90.67%) [33], Iran (69%) [23], Isiolo (38.2%) [26], Tanzania's two districts (3.5%) [27], and Mekelle (44.4%) [34]. The prevalence of isolated *S*. *aureus* was 16.7%, this finding was less than report of study conducted in Zambia (22%) [30], Iran (41.66%) [23], two studies conducted in Mekelle (27.5%) [32], and (26.7%) [34], and Hawassa (35.2%) [31]. However, this finding was greater than the reports of studies conducted in Tanzania (0.9%) [27] and Borena Abaya (7.29%) [13]. In addition, the prevalence of the third isolated bacteria, *Salmonella* spp., was 1.2%, that was less than reported in a study in Tanzania, Arusha (37.4%) [33] and Borena Abaya (11.5%) [13]. This variations among the prevalence could be due to sample collection, geographical area, enrolled samples' size, and the high proportion of those bacteria in milk related to the level of poor hygienic practices.

Multivariate analysis showed that milking without washing hands was nearly 2 times more likely to contaminate the milk and using unclean milk containers could contaminate raw milk nearly 7 times higher. This result was similar to studies carried out in Kampala [35], Hawassa [31], and Yabelo [22]. This may be due to the fact that the presence of bacteria on the hands and milk container goes through the milk and causes contamination.

Ampicillin was the drug highly resisted by (81.2%) *E*. *coli*. This finding is comparable with the report of previous studies [29, 36, 37]. Again, ampicillin was the drug mostly resisted by *Staphylococcus aureus* (64.3%) which means it is more comparable with the report of the previous study from Egypt [38] and Bangladesh [37] and less comparable with the report of Adgrat [29]. Similarly, ampicillin was highly resisted by isolated *Salmonella species*. This finding was more similar to study done in Bangladesh [37] compared to the study conducted in Jigjiga [21] and less similar to the study reported from Tanzania [36]. The higher resistance of ampicillin by isolated bacteria in the present study may be due to the communities' use of this drug as under-dose and extensively without prescription of antibiotics for the treatment of their diseases and also prolonged use for both prophylactic and therapeutic treatment.

An overall prevalence of multidrug resistance was 13.2%. The result of the present study was less as compared to report from Jigjiga (55.2%) [21]. Multidrug resistant (≥3 drugs) of isolated *S*. *aureus* were 28.6%. This result was less than the report from Egypt at 34.8% [38]. However, *S*. *aureus* multidrug resistance was less prevalent among studied milk samples. It is one obstacle to promoting health and life saving conditions for human beings. In addition, the isolated *Salmonella species* have shown higher multidrug resistance, particularly among commonly available drugs like tetracycline and ampicillin.

### 4.1. The Study Limitations

This study enrolled small sample size and also did not identify bacterial resistant gene to antimicrobials. The correlation between phenotype characteristics and genetic MDR has not been evaluated.

## 5. Conclusion and Recommendations

The results of this study revealed that raw milk is contaminated by total bacteria count and total coliforms count. The isolated bacteria from contaminated raw milk were *E*. *coli* 23 (30.7%), *Staphylococcus aureus* 14 (16.7%), and *Salmonella* spp. 1 (1.2%). Factors significantly associated with milk contamination were lack of handwash before milking and unclean milk containers. All bacteria isolated showed ampicillin resistance . Resistance against commonly used drugs among the communities might be difficult for medical care. On the basis of the results of this study, the following recommendations are made: the pastoral community must clean the milk containers properly and wash their hands before starting milking. Government and nongovernmental organizations must strengthen cooperation within the sector to use or build safe sources for the community. Regional and national governments must establish a diagnostic center to identify and monitor antibacterial resistance and prevent the animal transmission of pathogens. Doctors should consider drug resistance during the treatment of patients with diseases mediated by milk.

## Figures and Tables

**Table 1:** Sociodemographic characteristics and knowledge of milk owner Borena zone in Gomole district, Southern Ethiopia, 2019.

Variables	Category	Frequency	Percent
Sex of participant	Male	18	19
	Female	77	81

Age group	19–34 years	39	41.1
	35–50 years	40	42.1
	51–67 years	16	16.8

Educational status	Cannot read and write	84	88.4
	Can read and write	7	7.4
	Primary school	4	4.2

Train on milk handling	Yes	13	13.7
	No	82	86.3

Knowledge of milk borne disease	Yes	21	22.1
	No	74	77.9

**Table 2:** Hygienic practices, environment, transportation, and milk container characteristics among milk owners in the Borena zone Gomole district, southern Ethiopia, 2019.

Factors	Category	Frequency	Percent
Types of milk equipment	Plastic	72	75.8
	Aluminum/steel	3	3.2
	Wood	12	12.6
	Traditional pot	8	8.4

Source of water for cleaning	Tap water	63	66.3
	Well	18	18.9
	Spring	13	13.7
	Pond	1	1.5

Milk containers washing water	Cold water	20	21
	Hot water only	66	69.5
	Hot water + soap	9	9.5

Mode of transportation	On foot	35	36.8
	By motorcycle	52	54.7
	By animal	3	3.2
	By vehicle	5	5.3

Transportation hours	<1 hour	32	33.7
	1–2 hours	46	48.4
	2< hours	17	17.9

Mix milk from different sources	Yes	80	84.2
	No	15	15.8

Milk preserving method	By smoking	94	98.9
	By fridge	1	1.1

Clean barn practice	Daily	6	6.3
	Once a week	32	33.7
	Once a month	57	60

Hand wash before milking	Yes	10	10.5
	No	85	89.5

Udder wash practice	Yes	1	1.1
	No	94	98.9

**Table 3:** Observational findings among Borena zone Gomole District, South Ethiopia, 2019.

Factors	Category	Frequency	Percent
Personal hygiene	Looks poor	52	54.7
	Looks good	43	45.3

Cleanliness of milk container	Looks poor	77	81.1
	Looks good	18	18.9

Conditions around milk	Clean	13	13.7
	Dusty	16	16.8
	Cool conditions	41	41.2
	Hot conditions	25	26.3

**Table 4:** Total bacteria and coliform counts in raw milk from Gomole district, Borena zone, South Ethiopia, 2019.

Bacterial count	Mean ± SD (log_10_ CFU/mL)	Mean ± SD (no. CFU/mL)
TBC	7.57 ± 0.83	3.7 × 10^7^
TCC	6.51 ± 1.53	3.2 × 10^6^

TBC, total bacteria count; TCC, total coliform count; SD, standard deviation; CI, confidence interval.

**Table 5:** Proportion of contamination of milk and bacterial isolates among raw milk of Gomole district, Borena zone, South Ethiopia, 2019.

Bacteria isolates	From TBC contaminated milk (84)		From TCC contaminated milk (75)	
	No	%	No	%
*S*. *aureus*	14	(16.7)	—	—
*Salmonella Spp.*	1	(1.2)	—	—
*E*. *coli*	—	—	23	(30.7)

TBC, total bacteria count; TCC, total coliform count.

**Table 6:** Factors associated with contamination of raw milk from producers in Gomole district, Borena zone, South Ethiopia, 2019.

Factors	Category	Contaminated milk (no. %)	Noncontaminated milk (no. %)	COR (95% CI)	AOR (95% CI)
Gender	Male	17 (94.4)	1 (5.6)	2.54 [0.30–21.21]	
	Female	67 (87)	10 (13)	1	

Age group	19–34	34 (87.2)	5 (12.8)	2.21 [0.237–20.54]	
	35–50	35 (87.5)	5 (12.5)	2.14 [0.23–19.94]	
	51–67	15 (93.8)	1 (6.2)	1	

Educational status	Cannot read and write	76 (90.5)	8 (9.5)	0.32 [0.029–3.4]	
	Can read and write	5 (71.4)	2 (28.6)	1.20 [0.073–19.6]	
	Primary grade and above	3 (75)	1 (25)	1	

Source of water	Tape water	53 (84.1)	10 (15.9)	0.171 [0.21–1.40]^*∗*^	3.8 [0.395–36.7]
	Nontape water	31 (96.9)	1 (3.1)	1	1

Transportation hours	<1 hour	26 (81.2)	6 (18.8)	1.73 [0.31–9.68]	
	1–2 hours	43 (93.5)	3 (6.5)	0.52 [0.8–3.44]	
	2<hours	15 (88.2)	2 (11.8)	1	

Mix different milk	Yes	70 (87.5)	10 (12.5)	0.50 [0.039–4.23]	
	No	14 (93.3)	1 (6.7)	1	

Train on milk handling	Yes	11 (84.6)	2 (15.4)	0.69 [0.129–3.56]	
	No	73 (89)	9 (11)	1	

Knowledge of milk borne disease	Yes	17 (81)	4 (19)	0.44 [0.116–1.69]^*∗*^	0.69 [0.088–5.41]
	No	67 (90.5)	7 (9.5)	1	1

Hand wash before milking	Yes	4 (40)	6 (60)	0.042 [0.009–0.197]^*∗∗∗*^	2.4 [0.35–16.4]^*∗∗∗*^
	No	80 (94.1)	5 (5.9)	1	1

Personal hygiene	Looks poor	47 (90.4)	5 (9.6)	1.52 [0.431–5.39]	
	Looks good	37 (86)	6 (14)	1	

Cleanliness of milk containers	Looks poor	73 (94.8)	4 (5.2)	11.6 [0.53–63.4]^*∗∗∗*^	7.47 [0.0023–28.64]^*∗∗∗*^
	Looks good	11 (61.1)	7 (38.9)	1	1

Statistical significance at *P* < 0.01 = ^*∗∗∗*^, *P* < 0.05 = ^*∗∗*^and at *P* < 0.25 = ^*∗*^, COR = crude odds ratio; AOR = adjusted OR with CI = confidence interval.

**Table 7:** Drug sensitivity pattern of isolated bacteria.

Antimicrobials	*E. coli* (*n* = 23)		*S. aureus* (*n* = 14)		*Salmonella* spp. (*n* = 1)	
	S (%)	R (%)	S (%)	R (%)	S (%)	R (%)
Ampicillin	4 (21.1)	19 (79.9)	5 (35.7)	9 (64.3)	—	1 (100)
Ciprofloxacin	23 (100)	—	14 (100)	—	1 (100)	—
Chloramphenicol	23 (100)	—	10 (71.4)	4 (28.6)	—	2(100)
Gentamicin	23 (100)	—	13 (92.9)	1 (7.1)	1 (100)	—
Tetracycline	23 (100)	—	11 (78.6)	3 (21.4)	—	1 (100)

**Table 8:** Drug resistance pattern of isolated bacteria.

Bacteria strains	No. of isolates	Extensive drug resistant (XDR) no. (%)	Multidrug resistant (MDR) no. (%)	Pandrug resistant (PDR) no. (%)
*E*. *coli*	23	19 (82.6)	—	—
*S*. *aureus*	14	9 (64.3)	4 (28.6)	1 (7.1)
*Salmonella* spp.	1	—	1 (100)	—
Total	38	29 (76.3)	5 (13.2)	1 (2.6)

## Data Availability

Necessary data are available in the manuscript. Additional information can be received from corresponding author upon reasonable request..

## Supplementary Materials

Supplementary Materials S file 1: questionnaire and laboratory report form.docx. S file 2: bacteria isolation procedures.docx.
