# Supplementary material for: Assessment of Milk Contamination, Associated Risk Factors, and Drug Sensitivity Patterns among Isolated Bacteria from Raw Milk of Borena Zone, Ethiopia
Source: J Trop Med. 2022 Jun 20;2022:3577715. doi: 10.1155/2022/3577715 (PMC9236756; doi:10.1155/2022/3577715)
Supplement: Supplementary Materials — S file 1: questionnaire and laboratory report form.docx. S file 2: bacteria isolation procedures.docx. [file 3577715.f1.zip › 3577715.f1/S file 1. Questionnaire and Laboratory report form (1).docx]

**Questionnaire**

**Table 9.** Questionnaires administered to investigate factors associated with milk contamination

To be filled in space provided or make [√] at appropriate site

| No. | **Questions** | **Response** |
| --- | --- | --- |
| 1 | Sex of respondent | Male [ ] Female [ ] |
| 2 | How old are you? | ---------------------------- |
| 3 | What is your educational status? | 1. 1.Cannot read and write [ ] 2. 2.Can read and write [ ] 3. 3.Primary 1-8 [ ] 4. 4.Secondary 9-12 [ ] 5. 5.College or above [ ] |
| 4 | What is types of water you used to clean milk container? | 1. 1.Only with cold water [ ] 2. 2.Cold water with soap/ detergent [ ] 3. 3.Hot water only [ ] 4. 4.Wash with hot water and soap[ ] |
| 5 | What is your source of water used for cleaning purpose? | 1. 1.Tap water [ ] 2. 2.Pond [ ] 3. 3.Well [ ] 4. 4.Spring [ ] 5. 5.River [ ] |
| 6 | If source of water from pond or Well or Spring, is it? | 1. 1.Protected [ ] 2. 2.Un protected [ ] |
| 7 | What is the type of your milk utensils or equipment? | 1. 1.Plastic containers [ ] 2. 2.Aluminum/Stainless steel containers [ ] 3. 3.Wooden container[ ] 4. 4.Traditional pots [ ] 5. 5. Other (Specify)…………. |
| 8 | Are you ever trained on milk handling? | Yes [ ]  No [ ] |
| 9 | Do you know the diseases transmit through contaminated raw milk? | Yes [ ]  No [ ] |
| 10 | If yes mention the disease you know? | ……………………………. |
| 11 | Are you mix milk from different sources at one container | Yes [ ] No [ ] |
| 12 | How you preserve your milk? | 1.By smoking milk container [ ]  2.By store in fridge [ ] |
| 13 | How you transport your milk to reach the customers? | 1.On foot [ ]  2.by motorcycle [ ]  3.by animal [ ]  4.by vehicle [ ] |
| 14 | How many hours take for transportation? | 1. < 1 hour [ ]  2. 1-2 hours [ ]  3. 2 hours above [ ] |
| **Additional question for producers** | | |
| 15 | Do you wash your hands before milking? | Yes [ ] No [ ] |
| 16 | Do you wash udder before milking? | Yes [ ] No [ ] |
| 17 | How often you clean barn? | 1.Daily [ ]  2.Once a week [ ]  3.Once a month [ ] |
| **Observation checklist** | | |
| 18 | Personnel hygiene of milk producer and vendor | 1. 1. Looks poor [ ] 2. Looks good [ ] |
| 19 | General cleanliness of using containers | 1. Looks poor [ ] 2. Looks good [ ] |
| 20 | Condition around milk looks | 1. 1.Clean [ ] 2. 2.Dusty [ ] 3. 3.Cool (under shed) [ ] 4. 4.Hot (outside shed) [ ] 5. 5. Other (specify)…….. |

**Laboratory result form**

Lab serial number/ ID…………

Date and time of sample collection………………………

Result of total bacteria counted……………………………………….

Result of total coliform counted………………………………………

Identified bacteria *E. coli*, *S.aureus*, *Salmonella spp………………………………………………………*

Quality grade: poor/ good………………………..

**Table 1: Antimicrobial susceptibility testing result**

|  | Disk contents | Interpretation of the result (inhibition zone in mm) | | | | | | | | | | |
| --- | --- | --- | --- | --- | --- | --- | --- | --- | --- | --- | --- | --- |
|  |  | *Samonella* spp. | | | *E. coli* | | | | *S.aureus* | | |  |
|  |  | S | I | R | | S | I | R | S | I | R | Remark |
| Ampicillin | 10 μg |  |  |  | |  |  |  |  |  |  |  |
| Chloramphenicol | 30 μg |  |  |  |  | |  |  |  |  |  |  |
| Ciprofloxacin | 5 μg |  |  |  |  | |  |  |  |  |  |  |
| Gentamicin | 10 μg |  |  |  |  | |  |  |  |  |  |  |
| Tetracycline | 30μg |  |  |  |  | |  |  |  |  |  |  |

**S=** **sensitive** **I= intermediate R= resistant**
