# Supplementary material for: Assessment of Milk Contamination, Associated Risk Factors, and Drug Sensitivity Patterns among Isolated Bacteria from Raw Milk of Borena Zone, Ethiopia
Source: J Trop Med. 2022 Jun 20;2022:3577715. doi: 10.1155/2022/3577715 (PMC9236756; doi:10.1155/2022/3577715)
Supplement: Supplementary Materials — S file 1: questionnaire and laboratory report form.docx. S file 2: bacteria isolation procedures.docx. [file 3577715.f1.zip › 3577715.f1/S file 2. Bacteria isolation procedures (1).docx]

# TOTAL BACTERIA COUNT

## Standard methods agar (Plate Count Agar) (Oxoid, UK)

**Uses:** for total microbial plate count in milk and other materials of sanitary significance.

**Principles**

Uses Standard Methods Agar is recommended by APHA when enumerating bacteria of sanitary interest, which are indicators of contamination or microbial load in foods.

**Preparation procedures**

Suspend 23,5 grams of the medium in one liter of distilled water. Heat agitating frequently until boiling and completely dissolved. Dispense into appropriate containers and sterilize at 121 °C (15 lbs. sp) for 15 minutes.

**Inoculation and incubation**

In general, 1 ml. of the appropriate dilution is added to the sterile agar at a temperature of 44-45°C, mixed gently and poured into sterile Petri dishes. Incubate the Petri dishes at incubated at 37 ºC for 24 hours and count the developed colonies. Consult the specific texts of APHA for the particular sample applications.

## Peptone Water (Oxoid, UK)

**Uses**

A diluent for the homogenization of samples in microbiological analysis of food.

**Principles**

Recommended as a diluent for the homogenization of food samples containing suspected contaminants such as Salmonella, etc. Changes in pH may cause damages to bacteria growth. This media maintains a high pH due to its phosphates content. This medium complies with the recommendations of the International Standard Organization ISO (1933) and the German DIN Regulations 10181 and 10160 for the examination of milk, meat and meat products.

**Preparation procedures**

Dissolve 25,5 grams of the medium in one litre of distilled water. Mix well. Distribute into appropriate containers and sterilize at 121°C (15 lbs. sp.) for 15 minutes. One mL of raw milk sample was added into a sterile test tube containing 9 mL of sterile peptone water. Then serial dilutions were made by transferring 1 ml of the previous dilution in 9 ml of sterile peptone water in 11 duplicates.

**Total bacteria and total coliform count quality determination**

**Table 1: Ethiopia/East Africa standard of total bacteria count in raw milk grade**

| Total bacteria count per ml | Logarithm (Log_10_) | Grade | Quality |
| --- | --- | --- | --- |
| < 200,000 | <5.3 | Ⅰ | Very good |
| 200,000-1000,000 | 5.3-6 | Ⅱ | Good |
| 1000,000-2000,000 | 6-6.3 | Ⅲ | Fair |
| >2000,000 | >6.3 | Ⅳ | Poor |

# Total coliform count (TCC)

## Eosin Methylene Blue (E.M.B) Agar (Oxoid, UK)

**Uses**

For the isolation and differentiation of coliforms from other enterobacteria of medical and sanitary interest

**Principles**

It is widely used in medical bacteriology, in techniques recommended by the APHA and for the detection and enumeration of coliform microorganisms, which can contaminate foods and drinking water. Due to the lactose and sucrose, the medium can be differential in primary culture: salmonellas and shigellas which are lactose-negative can be differentiated from other lactose- negative but sucrose-positive organisms such as Proteus vulgaris, Citrobacter and Aeromonas.

The accompanying microflora which hinders the isolation of medically important organisms are inhibited by the dyes in the formula, especially gram-positives. It can also be used for the rapid identification of C. albicans (incubated in CO_2_) and sometimes to isolate.

**Preparation procedures**

Suspend 36 grams of the medium in one litre of distilled water. Mix well. Heat with frequent agitation and boil for one minute. Sterilize in autoclave at 121°C (15 lbs. sp.) for 15 minutes. Cool to 45-50°C. Swirl gently, avoiding the formation of bubbles and pour into Petri dishes.

**Characteristics of the colonies**

Nocardia. E. coli Elevated or slightly convex. 2-3 mm. in diameter, with transmitted light blue-black center with a narrow, clear edge. Blue-green metallic sheen with reflected light. Some strains show no metallic sheen. Small tendency to confluent growth. E. aerogenes Klebsiella Large colonies, 4-6 mm. in diameter, mucoid with a tendency to run together. Usually no metallic sheen. With transmitted light, gray-brown centers with clear edges. Salmonella Shigella Slightly elevated, medium size 1-2 mm. in diameter. Transparent, from colourless to amber. C. albicans Feathery, spider-like colony after 24-48 hours incubation in CO_2_ at 35-37°C. Never presents a typical colonial appearance. Coagulase-positive staphylococci Very small punctiform, colourless and inhibited. Proteus species when there is no swarming, similar to Salmonella and Shigella. Swarming can be minimized by adding a very small amount of alpha-p-nitrophenyl- glycerol.

**Table 2: East Africa standard of total coliform count in raw milk grade**

| Total coliform count per ml | Logarithm (Log_10_) | Quality |
| --- | --- | --- |
| 0-1000 | 0-3 | Very good |
| 1000-50,000 | 3-4.7 | Good |
| >50,000 | >4.7 | Poor |

# Bacteria Isolation and Identification

**Ⅰ) Isolation and Identification of E. coli**

From coliforms poor graded culture plates, typical colonies were subjected to Gram staining to study the staining properties and cellular morphology and at the same time it was inoculated on to sterile Nutrient Agar for biochemical tests. In general Coliform organisms was identified based on Gram staining reaction, growth characteristics on EMBA (pigmented colony) urease and production of H**_2_**S, motility and indole test on SIM (Sulfide, Indole, Motility) a tubed semisolid agar medium, sugar fermentation patterns on Triple Sugar Iron Agar (TSI) tests (Cheesbrough, 2006).

## A) Manufacturer instructions of biochemical Tests

**i) SIM Medium** (Oxoid, UK)

**Principles**

A motility-indole medium combined with sulphide-production in one tube is helpful in the identification of the *Enterobacteriaceae*. The production of H_2_S is a useful diagnostic test in the identification of enteric bacteria and is helpful in the differentiation between *Salmonella* and *Shigella*. The sulphate-reducing bacteria will produce H**_2_**S and further chemical substitution results in ferrous sulphide being formed along the line of inoculation.

The production of indole from tryptophan is one of the diagnostic tests used in identifying enteric bacteria. Tryptone is incorporated into the medium since it is a tryptophan-rich peptone, and after incubation, indole can be identified by a red dye complex reaction with one of several reagents.

- The use of only 0.35% agar in the medium results in the production of a semi-solid medium, ideal for the examination of motility.
- SIM Medium is therefore designed to determine three characteristics: H**_2_**S production, indole production and motility.

**Instructions**

Suspend 30 g in 1 litre of distilled water and boil to dissolve the medium completely. Dispense into final containers and sterilize by autoclaving for 15 minutes at 121°C.

**Procedure**

- The medium should be dispensed in tubes or bottles and when cool, inoculated once with a pure culture, by inserting a straight wire to about one-third of the depth of the medium and incubated at 35°C for 18 hours or longer, if necessary, and examined for motility, H**_2_**S production and finally indole production from tryptophan.

**Procedure for Indole test:**

1. Add 0.2 ml of Kovac’s Reagent (consists of amyl alcohol, para-dimethyl amino-benz-aldehyde and concentrated HCl) or Ehrlich’s reagent which contains 4 (p)-dimethylaminobenzaldehyde to the tube and allow standing for 10 minutes.
2. A dark red color in the reagent constitutes a positive indole test. No change in the original colour of the reagent constitutes a negative test.

**Motility test**

Non-motile organisms grow only along the line of inoculation, whereas motile species show either a diffuse even growth spreading from the inoculum, turbidity of the whole medium, or more rarely, localized outgrowths which are usually fan-shaped or occasionally nodular. H**_2_**S production is shown by blackening of the line of inoculation.

Precautions: To avoid delay in initiating growth always sub-culture from solid media.

**ii) Triple Sugar Iron (TSI) Agar** (Oxoid, UK)

**Principle**

It is a composite medium for the differentiation of *Enterobacteriaceae* according to their ability to ferment lactose, sucrose and glucose, and to produce H**_2_**S. These organisms may ferment lactose slowly or not at all. Some *Proteus* and other species may give similar reactions to *Salmonellae* and it is necessary to distinguish them by their ability to hydrolyze urea. For this reason TSI Agar should be used in parallel with Urea.

This medium was formerly considered to be interchangeable with Kligler medium for the detection of H**_2_**S producing *Enterobacteriaceae*. It is now thought that TSI Agar is not suitable for the detection of H**_2_**S production by sucrose-fermenting organisms, such as some *Citrobacter* and *Proteus* species, in which the sucrose fermentation masks the H**_2_**S indicator in the medium.

**Instructions**

Suspend 65 g in 1 litre of distilled water. Bring to the boil to dissolve completely. Mix well and distribute. Sterilize by autoclaving at 121°C for 15 minutes. Allow the medium to set in sloped form with a butt about 1 inch deep.

**Procedures**

1. Pick a single colony from the surface of selective plating medium and inoculate two separate tubes of media from one single isolated colony:

- TSI Agar – smear the slope and stab the butt.
- Urea Broth Base with added Urea Solution.

1. Incubate at 35°C.
2. Examine the Urea Broth tube after 5h and again after 18h incubation. Discard tubes showing a red or pink coloration. Where there is no urea hydrolysis, examine the TSI Agar tubes after 18h and 48h.

**iii) Urea Agar Base** (Oxoid, UK)

An agar base for the preparation of Christensen’s medium to detect rapid urease activity of the and non-rapid urease activity of some *Enterobacteriaceae*.

**Instructions**

Suspend 2.4 g in 95 mL of distilled water. Bring to the boil to dissolve completely. Sterilize by autoclaving at 115°C for 20 minutes. Cool to 50°C and aseptically introduce 5 mL of sterile 40% Urea Solution SR0020. Mix well, distribute 10 ml amounts into sterile containers and allow to set in the slope position.

**Principle**

Urea Agar Base is recommended for the preparation of Christensen medium for the detection of rapid urease activity of the urease-positive *Proteae*. The urea medium may be used for the detection of urea hydrolysis by some other *Enterobacteriaceae* but the incubation period is much longer, 24-48h.

**Procedures**

1. Heavily inoculate the surface of a Urea Agar slope with a pure culture of the organism to be tested.
2. When inoculated with urease-positive Proteae the reaction is usually complete after 3-5 h at 35°C: urease producing organisms hydrolyze the urea to form ammonia, and the medium changes from orange to pink. 40% Urea Solution SR0020 is supplied, as a sterile solution in ampoules, for the convenient preparation of this medium.

**iv) Methyl Red** The Methyl Red Test is a component of the IMViC battery of tests (Indole, Methyl Red, Voges-Proskauer, and Citrate) used to differentiate the Enterobacteriaceae. It identifies bacterial ability to produce stable acid end products by means of a mixed-acid fermentation of glucose.

**Principle**

To test the ability of the organism to produce and maintain stable acid end products from glucose fermentation and to overcome the buffering capacity of the system.This is a qualitative test for acid production. Red color is the only true indication of a positive result.

Material required

Test tube, methyl red reagent, Inoculating loops, Bunsen burner, Incubator

**Procedures**

• Inoculate the MR/VP broth with a pure culture of the test organism and

• Incubate at 35°for 48 to 72 hrs.

• Add 5 drops of MR reagent to the broth and observe colour change

- The culture color change to red indicate positive

**v) Citrate Agar**

**Principle**

A Simmons Citrate Agar is often part of a battery of tests used to identify *Enterobacteriaceae* based on the utilization of citrate as the sole source of carbon. The medium is virtually a solidified form of Koser citrate medium. The addition of bromothymol blue indicator to the medium was a distinct improvement.

**Instructions**

Suspend 23 g in 1 litre of distilled water. Bring to the boil to dissolve completely. Sterilize by autoclaving at 121°C for 15 minutes.

The medium may be used either as slopes in test tubes or as a plate medium in Petri dishes. In both cases the surface of the medium is lightly inoculated by streaking Incubation for 48 hours at 35°C is recommended. Positive growth (i.e. citrate utilization) produces an alkaline reaction and changes the colour of the medium from green to bright blue, whilst in a negative test (i.e. no citrate utilization) the colour of the medium remains unchanged. *Escherichia coli* including serotypes from epidemic infantile enteritis. Simmons Citrate Agar may be used to differentiate citrate-positive *Salmonella enteritidis* and members of *Salmonella* subgenus II, III and IV from the citrate-negative *Salmonella typhi*, *Salmonella paratyphi A*, *Salmonella pullorum* and *Salmonella gallinarum*.

**Precautions**

It is important not to carry over any nutrients into the citrate medium because this will result in false positive tests. Dilute the inoculum in saline before inoculating the citrate medium to avoid a carry-over of other carbon source.

**Ⅱ) Isolation and identification of *salmonella***

From TBC poor graded samples a portion of 1 ml of milk was pre-enriched in 9 ml of lactose broth at 37°C for 24h. Then, 1 ml of pre-enrichment sample was inoculated in to 10 ml selenite broth and incubated at 37°C for 24 hr. A loop full of selective enrichments were streaked on Xylose- lysine decarboxylase (XLD), and incubated at 37°C for 24 hrs. All suspected non-lactose fermenting salmonella colonies were picked from plate agars and streaked onto nutrient agar plate and then incubated at 37°C for 24 hrs. From each plate agar pure isolate single colony was picked and inoculated into biochemical tubes for biochemical tests.

Identification of suspect *salmonella* isolates using MIU medium, Citrate, TSI and LIA as procedures mentioned above and incubate at 35-37°C overnight. Examine biochemical depending on color changes and gas production as characteristics of *Salmonella* spp*.* throughout the medium.

# i) Selenite cystine F broth

**Uses:** For the selective enrichment of Salmonella and some other Shigella strains

**Principles**

The color of medium should be beige to pale pink. Uses In 1953, North and Bartram modified an enriched medium prepared by adding the amino acid cystine. This amino acid establishes a redox potential that seems to be very good for enrichment and recovery of Salmonella and some strains of Shigella, present in limited numbers in feces, diverse foods, and other products of sanitary concern. Selenite Cystine Broth is used particularly to limit the loss of sensitivity that affects other enrichment media especially in food products with a high content of organic material, for example, foods of egg and egg powder. Selenite Cystine Broth inhibits the early multiplication of bacteria such as coliforms, but allows the salmonellas to grow with ease.

**Preparation procedures**

Suspend 23 grams of the medium in one liter of distilled water. Mix well and heat slowly until the medium is dissolved. Dispense in screw-capped test tubes sterilize under flowing steam for 5 minutes. Do not autoclave.

**Inoculation and incubation**

Nevertheless, after 18 hours of incubation, the commensal microorganisms rapidly increase and begin to impede the isolation of salmonellas, so that it is necessary to re-streak or subculture before the elapse of this critical time. These inoculations to differential solid media should be performed at the end of incubated at 37°C for 24 hours.

# ii) Xylose Lysine Desoxycholate (XLD) Agar

**Uses**: for the isolation of enter pathogenic bacteria, especially from the genera of Shigella, Salmonella, and Arizona

**Principles**

In XLD Agar it is possible to obtain the following differential this medium was developed principally for isolating Shigella and Providencia. It has been shown to be more effective than other enteric differential media, reactions: the degradation of xylose, lactose and sucrose, with the production of acid, manifested in the color change from red to yellow. Sodium thiosulfate serves as a reactive substance with the iron salt as an indicator of the formation of hydrogen sulfide. The bacteria that decarboxylate the lysine to cadaverine are identified by the presence of a purple-red color around the colonies due to the elevation of pH.

**Preparation procedure**

Suspend 55 grams of the medium in one liter of distilled water. Heat with frequent agitation until a temperature of approximately 90ºC. Do not boil. Transfer immediately into a water bath at about 50ºC. Pour into Petri plates as soon as it has cooled. The medium should have a reddish color and be clear, or almost clear. Excessive heating or a prolonged stay in the water bath produces precipitation. When this occurs, reactions are satisfactory, but colonies may be slightly smaller. This precipitation can be eliminated by paper filtration.

**Characteristics of the colonies** Salmonella: Red, transparent, yellow edges with black centers only if H_2_S is produced after incubated at 37°C for 24 hours.

**iii) Nutrient agar**

**Uses**: for the cultivation and enumeration of non-fastidious organisms in food, water, faeces and other materials

**Principles**

Nutrient Agar is a general purpose medium, not selective but suitable for the cultivation of non-fastidious microorganisms. It can be used as a colony count medium in sanitation, medical, and industrial bacteriology. There are many uses for Nutrient Agar in the bacteriological analysis of drinking water, waste water, milk and other foods. It is also used in the multiplication of microorganisms to produce vaccines and antigens in general; in the tests of sensitivity and resistance, and as a base to prepare an enriched medium by adding ascitic fluid, etc. It is used in biochemical test, for example indole decarboxylase and lysine decarboxylase.

**Preparation procedures**

Suspend 23 grams of the medium in one liter of distilled water. Mix well and leave to stand until the mixture is uniform. Heat with gentle agitation and boil for one or two minutes, or until completely dissolved. Dispense and sterilize at 121°C (15 lbs.sp) for 15 minutes.

**iv) Lysine Iron Agar (LIA)**

**Instructions**

Suspend 34 g in 1 litre of distilled water. Bring to the boil to dissolve completely. Dispense into tubes and sterilize by autoclaving at 121°C for 15 minutes. Cool the tubes in an inclined position to form slants with deep butts.

**Principle**

Lysine Iron Agar is a differential medium which detects salmonellae (including lactose-fermenting *Salmonella arizonae*) by lysine decarboxylase activity and H**_2_**S production.

Further, many of these cultures, when transferred to TSI Agar slants, produced acid conditions in the medium so quickly that the expected positive reaction for H**_2_**S was suppressed. Since *Salmonella arizonae* strains which ferment lactose rapidly are found occasionally in outbreaks of food infection, it is important to determine their occurrence. The only recognized groups of *Enterobacteriaceae* which regularly decarboxylate lysine rapidly and produce large amounts of H**_2_**S, are the *Salmonellae*. Lysine Iron Agar is therefore a sensitive medium for the detection of lactose-fermenting and non-lactose fermenting *salmonellae*.

**Procedures**

1. The medium is tubed, sterilized and slanted so that a short slant and deep butt are formed. It is inoculated with a straight needle by stabbing to the base of the butt and streaking the slant. The caps of the tubes must be replaced loosely so that aerobic conditions prevail on the slant.
2. Incubate at 35°C overnight. Cultures which rapidly produce lysine decarboxylase cause an alkaline reaction (purple colour) throughout the medium. Those organisms that do not decarboxylate lysine produce an alkaline slant and an acid butt (yellow colour). Cultures which produce H**_2_**S cause an intense blackening in the medium.

**Table 3: Key biochemical isolation characteristics for *E. coli* and *Salmonella***

| Genus | Sugar fermentation | | | | Gas | MR | VP | I | C | Mo |
| --- | --- | --- | --- | --- | --- | --- | --- | --- | --- | --- |
|  | G | L | S | M |  |  |  |  |  |  |
| *Escherichia coli* | + | + | + | + | + | + | - | **+/-** | **-** | **-** |
| *Salmonella* spp | + | - | - | + | + | + | - | **+** | **-** | **+** |

G: Glucose, L: Lactose, S: Sucrose, M: Maltose MR: Methyl Red, VP: Voges Proskauer I: Indole, C: Citrate, U: Urease H2S: Hydrogen Sulphide Mo: Motility

Adapted from: Ethiopian Health and Nutrition Research Institute (Food Microbiology Laboratory manual, 2003)

**Ⅲ) Isolation and Identification of *S. aureus***

Mannitol Salt Agar was used to isolate *Staphylococcus aureus* based on colony appearance (golden yellow pigment production) and was re cultured on Nutrient Agar to be identified based on gram staining, catalase and coagulase tests.

**i) Mannitol salt agar** (Oxoid, UK)

**Uses:** for the isolation of pathogenic Staphylococci.

**Principles**

This is a selective medium prepared according to the recommendations of Chapman for the isolation of presumptive pathogenic staphylococci. Most of the other bacteria are inhibited by the high concentration of salt. The degradation of mannitol with the production of acid changes the color of the medium from rose to yellow. Due to its high content of sodium chloride, a heavy inoculum of the material in study can be used.

**Preparation procedures**

Suspend 111 grams of the medium in one litre of distilled water. Mix well and heat with frequent agitation until complete dissolution. Boil for one minute. Sterilize in autoclave at 121°C (15 lbs. of steam pressure) for 15 minutes. Pour into Petri dishes.

**Characteristics of the colonies**

Generally the plates are incubated for 37°C for 24 to 48 hours, colonies of non-pathogenic staphylococci appearing as small colonies surrounded by a red or purple zone. The mannitol fermenting pathogenic staphylococci are larger and are surrounded by a yellow zone.

**ii) Gram staining**

**Principle**

The principle of Gram’s stain is that cells are first fixed to slide by heat and stained with a basic dye (e.g. **Crystal violet)** acts as the **primary stain**. This stain is used first and stains all cells purple. **Grams iodine** reagent serves as a mordant, a substance that forms an insoluble complex by binding to the primary stain. The resultant crystal violet-iodine (CV-I) complex serves to intensify the colour of the stain, and all the cells will appear purple-black at this point.

In gram-positive cells, this CV-I complex binds to the magnesium-ribonucleic acid component of the cell wall.

**Procedure of Gram’s Stain**

 1. Fix the dried smear with heat by gently passing it over sprit lamp or Bunsen burner.

2. Cover the fixed smear with crystal violet stain for 30 – 60 seconds

3. Rapidly wash off the stain with clean water

4. Tip of all the water, and cover the smear with Lugol’s iodine for 30 – 60 seconds

5. Wash off the iodine with clean water

6. Decolorize rapidly with acetone-alcohol for 30 seconds and wash immediately with clean water.

7. Cover the smear with Neutral red or Safranin for 2 minutes

8. Wash off the stain with clean water and wipe the back of the slide clean, and place it in a draining rack for the smear to air dry

10. Examine the smear microscopically, first with the 40x objective to check the staining and to see the distribution of material, and then with oil immersion objective to report the bacteria and cells.

Results interpretation

- Gram positive bacteria …..………………….. Purple(blue)
- Gram negative bacteria …….……………….. Pale to red

**iii) Catalase**

**Principle**: it tests the ability of the organism to liberate O_2_ from H_2_O_2_ by the action of catalase.

**Procedures:**

1. A small amount of growth from pure culture will be placed onto a clean microscope slide.
2. Then few drops of H_2_O_2_ added and mixed with a toothpick.

- A positive result is indicated by rapid evolution of O_2_ as evidenced by bubbling.
- If no bubble the test is negative.

1. This test is used to differentiate those bacteria that produce the enzyme catalase, such as staphylococci, from non-catalase producing bacteria such as streptococci.

**iv) Coagulase test**

**Principle**:

This test is used to differentiate *Staphylococcus aureus*(positive) from coagulase negative Staphylococci. S. aureusproduces two forms of coagulase: bound and free.  ‐Bound coagulase or clumping factor, is bound to the bacterial cell wall and reacts directly with fibrinogen. When a bacterial suspension is mixed with plasma, this enzyme causes alteration in fibrinogen of the plasma to precipitate on the staphylococcal cells, causing the cells to clump. ‐Free coagulase is produced extracellularlyby the bacteria that causes the formation of a clot when S. aureuscolonies are incubated with plasma.

**Method:**

**a) Slide test**: (for bound coagulase)

- Place a drop of coagulase plasma on a clean, dry glass slide.
- Place a drop of distilled water or saline next to the drop of plasma as a control.
- With a loop or  wooden stick, emulsify a portion of the isolated colony being  tested in each drop
- Mix well and rock the slide gently for 5 to 10 seconds.

**b) Tube test**: (for free coagulase)

Emulsify several colonies in 0.5 ml of rabbit plasma (with EDTA) to give a milky suspension. Incubate tubes at 35^o^C in ambient air for 4 hrs. Check for clot formation.If negative at 4 hrs, incubate at room temperature overnight and check again for clot formation.

**Reading Results**:

A. Slide test: ‐Positive: Macroscopic clumping in 10 seconds

‐Negative: No clumping in either drop. ‐Note:  All negative slide tests must be confirmed using the tube test.

B. Tube test: ‐Positive: Clot of any size

-Negative: No clot

Coagulase Positive: *Staphylococcus aureus,* Coagulase negative: *Staphylococcus epidermidis*

## B) Antimicrobial Sensitivity Test

**Method:** Kirby-Bauer disk diffusion

**Principle:** the antibiotics will diffuse in a radial manner from the disc and will inhibit bacterial growth around it.

**Procedure** Make sure the turbidity is equivalent to 0.5 McFarland and the thickness of the Mueller Hinton agar is 4 mm.

1. Using a sterile wire loop, touch 3 – 5 well – isolated colonies of similar appearance to the test organism and emulsify in 3 – 4 ml to sterile physiological saline or nutrient broth. In a good light match the turbidity of the suspension to the turbidity standard (mix the standard before use)
2. Using a sterile swab, inoculate a plate of Muller Hinton agar. Remove excess fluid by pressing and rotating the swab against the side of the tube above the level of the suspension.
3. Streak the swab evenly over the surface of the medium in three directions rotating the plate approximately 60^o^ to ensure even distribution. With the Petri dish lid in place, allow 3 – 5 minutes for the surface of the agar to dry. Using sterile forceps or multi disc dispenser, place the appropriate antimicrobial discs evenly distributed on the inoculated plate.
4. Within 30 minutes of applying the discs, invert the plate and incubate it aerobically at 35^o^C for 16 – 18 hours. After overnight incubation, examine the control and the test plates. Using a ruler measure the diameter of each zone of inhibition in mm on the underside of the plate. the end point of inhibition is where growth starts.

**Purpose:** to detect the *in vitro* relationship between an organism and an antibiotic and predict antibiotic resistance pattern in the community.

Zone sizes will be interpreted using manufacturer inserted leaflet and CLSI recommendation as shown in table below (CLSI, 2018).

**Table 4: Antimicrobial agents with zone diameter interpretive standards**

| **No.** | Antimicrobial agents | Disk content | Zone diameter interpretive criteria (nearest mm) | | |
| --- | --- | --- | --- | --- | --- |
|  |  |  | Sensitive | Intermediate | Resistant |
| **1** | Ampicillin | 10 μg | ≥14 | 12-13 | ≤11 |
| **2** | Chloramphenicol | 30 μg | ≥18 | 13-17 | ≤12 |
| **3** | Ciprofloxacin | 5 μg | ≥21 | 16-20 | ≤15 |
| **4** | Gentamicin | 10 μg | ≥15 | 13-14 | ≤12 |
| **5** | Tetracycline | 30 μg | ≥15 | 12-14 | ≤11 |

**Sensitive (S)** – the susceptible category implies that isolates are inhibited by the usual achievable concentration of antimicrobial agent when the recommended dosage is used for the site of infection.

**Intermediate (I)** – a category defined by a breakpoint that includes isolates with or zone diameters within the intermediate range which response rates lower than for susceptible isolates.

**Resistant (R)** – the resistant category implies that isolates are not inhibited by the usually achievable concentrations of the agent with normal dosage schedules, and that demonstrate zone diameters fall in the range where specific microbial resistant mechanisms (understood from CLSI, 2018).
